# *Punica granatum L.* Polyphenolic Extract as an Antioxidant to Prevent Kidney Injury in Metabolic Syndrome Rats

**DOI:** 10.1155/2023/6144967

**Published:** 2023-01-05

**Authors:** Anna Radajewska, Jakub Szyller, Joanna Niewiadomska, Agnieszka Noszczyk-Nowak, Iwona Bil-Lula

**Affiliations:** ^1^Division of Clinical Chemistry and Laboratory Hematology, Department of Medical Laboratory Diagnostics, Faculty of Pharmacy, Wroclaw Medical University, Borowska 211A, 50-556 Wroclaw, Poland; ^2^Department of Internal Medicine and Clinic of Diseases of Horses, Dogs and Cats, Faculty of Veterinary Medicine, University of Environmental and Life Sciences, Grunwaldzki Square 47, 50-366 Wrocław, Poland

## Abstract

**Introduction:**

Obesity and metabolic syndrome (MetS) constitute a rapidly increasing health problem and contribute to the development of multiple comorbidities like acute and chronic kidney disease. Insulin resistance, inappropriate lipolysis, and excess of free fatty acids (FFAs) are associated with glomerulus hyperfiltration and atherosclerosis. The important component of MetS, oxidative stress, is also involved in the destabilization of kidney function and the progression of kidney injury. Natural polyphenols have the ability to reduce the harmful effect of reactive oxygen and nitrogen species (ROS/RNS). Extract derived from *Punica granatum L.* is rich in punicalagin that demonstrates positive effects in MetS and its associated diseases. The aim of the study was to investigate the effect of bioactive substances of pomegranate peel to kidney damage associated with the MetS.

**Methods:**

In this study, we compared biomarkers of oxidative stress in kidney tissue of adult male Zucker Diabetic Fatty (ZDF) rats with MetS and healthy controls that were treated with *Punica granatum L.* extract at a dose of 100 or 200 mg/kg. Additionally, we evaluated the effect of polyphenolic extract on kidney injury markers and remodeling. The concentration of ROS/RNS, oxLDL, glutathione (GSH), kidney injury molecule-1 (KIM-1), neutrophil gelatinase-associated lipocalin (NGAL), metalloproteinase 2 and 9 (MMP-2, MMP-9), and the activity of superoxide dismutase (SOD) and catalase (CAT) were measured.

**Results:**

The data showed significant differences in oxidative stress markers between treated and untreated MetS rats. ROS/RNS levels, oxLDL concentration, and SOD activity were lower, whereas CAT activity was higher in rats with MetS receiving polyphenolic extract. After administration of the extract, markers for kidney injury (NGAL, KIM-1) decreased.

**Conclusion:**

Our study confirmed the usefulness of pomegranate polyphenols in the treatment of MetS and the prevention of kidney damage. However, further, more detailed research is required to establish the mechanism of polyphenol protection.

## 1. Introduction

Metabolic syndrome (MetS) and obesity are an epidemic and are huge medical problems throughout the world. About one-third of US adults suffer from MetS [1]. In Europe, MetS has in general a 24.3% prevalence (23.9% in men and 24.6% in women) that increases with age (3.7% in people aged 20–29 years to >30% in those 70 years and older) [2]. MetS, also known as insulin resistance syndrome or syndrome X, is a cluster of risk factors for the development of renal disease, atherosclerotic cardiovascular disease, type 2 diabetes mellitus, and stroke [3–6]. It is defined as glucose intolerance, hypertension, dyslipidemia, and central (intra-abdominal) obesity with insulin resistance and a proinflammatory state [7–9]. MetS is present if three or more of the following criteria are met: waist circumference over 102 cm (40 inches, men) or 89 cm (35 inches, women), blood pressure over 130/85 mmHg, fasting triglyceride level over 150 mg/dL, fasting high-density lipoprotein (HDL) cholesterol level less than 40 mg/dL (men) or 50 mg/dl (women), and fasting blood sugar above or equal 100 mg/dL [8]. Obesity (especially visceral) plays an important role in the complex pathomechanism of MetS and raises the risk of chronic kidney disease (CKD) by 4-fold [10]. The most widely accepted hypothesis for the underlying pathophysiology of MetS is that insulin resistance is a consequence of visceral obesity, inappropriate lipolysis, excess of free fatty acids (FFAs), and the action of adipokines [11]. FFAs are produced during the hydrolysis of fats and released from adipose tissue during lipolysis. The insulin resistance of adipose tissue impairs the insulin-mediated inhibition of lipolysis, leading to an increase in circulating FFAs that subsequently inhibit the effects of insulin. Lipids and FFAs accumulation in the muscle and insulin resistance result in a reduction in glycogen synthesis and glucose transport due to the inhibition of insulin-dependent glucose uptake. The liver esterifies the fatty acids to triglycerides and secretes them as very low-density lipoproteins (VLDLs). Additionally, fatty acids promote the increased production of glucose and lipogenesis. In the setting of insulin resistance, the rate of lipolysis will increase, resulting in increased fatty acid production [11, 12]. Insulin resistance also causes an increase in serum viscosity which contributes to an increased risk of cardiovascular diseases [12]. Also, there is a positive correlation between fat accumulation, oxidative stress, and inflammation. Obesity and insulin resistance are associated with increased activity of nicotinamide adenine dinucleotide phosphate (NADPH) oxidase leading to the production of reactive oxygen species (ROS) [13]. ROS have many effects related to LDL oxidation, endothelial damage, platelet aggregation, and activation of proinflammatory factors, which initiate a vicious cycle of chronic inflammation, endothelial damage, and fibroblast proliferation that further contributes to the development of hypertension, dyslipidemia, diabetes, and cardiovascular diseases [14]. Patients with MetS have a 2–3 fold higher risk of increased albumin excretion [15]. Thus, MetS is an important predictor of early renal dysfunction [16]. This information provides an essential rationale to verify whether components of MetS can prevent the development and progression of renal damage. CKD is a significant public health problem and is one of the major risk factors for cardiovascular disease (e.g., acute myocardial infarction, heart failure, and arrhythmias) and premature death [17, 18]. MetS may also contribute to the development of acute kidney injury (AKI) [19]. AKI is defined as a sudden decrease in kidney function. It is a broad clinical syndrome encompassing various etiologies, including specific kidney diseases and nonspecific conditions (e.g., toxic injury and ischemia) as well as extrarenal pathology. Criteria for AKI include an increase in serum creatinine (sCr) by ≥50% within 7 days, an increase in sCr by ≥0.3 mg/dL (26.5 *μ*mol/L) within 2 days, or oliguria for ≥6 hours [20]. The major signaling pathways involved in AKI, among others, are the renin-angiotensin-aldosterone (RAA) axis, tumor necrosis factor alpha (TNF-*α*), transforming growth factor beta (TGF-*β*), NF-*κ*B, hypoxia inducing factor (HIF1*α*), and oxidative stress similar to MetS [21]. Oxidative stress and lipid peroxidation, characteristics for MetS, correlate with the biomarkers of glomerular and tubular damage in AKI and are the main pathophysiological factor in the initiation and progression of AKI through depletion of proximal renal tubular antioxidant capacity and induction of free radicals, leading to tubular and epithelial cell dysfunctions [22]. ROS may also influence hemodynamics and inflammation. Inflammatory process and infiltrating leukocytes with high levels of NADPH oxidase activity represent a further source of superoxide during AKI [23]. A role of xanthine oxidase activity in AKI has also been suggested, but the results are not conclusive. Oxidative stress as an consequence of mitochondrial dysfunction and mitochondrial electron transport chain dysfunction during MetS, may represent significant source of ROS contributing to AKI. Also, hypoxic environment reduces renal SOD expression and increases sensitivity to AKI [23].

Adipose tissue is not only a fat reservoir, but it is also a site of adiponectin, leptin, resistin, angiotensin-II (ANG-II), TNF-*α*, and TGF-*β* production [24]. The pathophysiology of CKD during the course of MetS is very complex, and the relationship is bidirectional. Injury of podocytes, an increase in glomerular size, and sclerosis of the mesangium are early and key manifestations of MetS and nephropathy in rat models [25]. In humans, obesity is associated with CKD, glomerulomegaly, and proteinuria, which is the first clinical symptom [19, 20, 24–26]. Obesity-related increases in renal tubular sodium reabsorption, hyperfiltration (associated with increased blood pressure), sympathetic nervous system activation, hyperleptinemia, hyperinsulinemia, fatty acids levels, ANG-II, and baroreceptor reflex alterations result in kidney damage and reduced glomerular filtration rate (GFR) [26–28]. An excess of fatty acids leads to a compensatory increase in oxidation and release of lipid peroxidation products [29, 30]. These compounds can lead to apoptosis and fibrosis [31, 32]. Systemic oxidative stress is considered to be the most important underlying pathophysiologic mechanism of MetS. Thus, oxidative stress can be an important link between MetS and kidney damage.

Polyphenols, derived from plants, represent a very large group of at least 10,000 different compounds that possess aromatic rings, one or more ligand groups, and antioxidant properties [33, 34]. Polyphenols can reduce fat deposition via the *γ*-adiponectin pathway [35], reduce the prevalence of coronary heart disease [36], significantly improve fasting insulinemia, decrease the homeostatic model assessment of insulin resistance (HOMA-IR), and reduce the levels of blood glucose and hemoglobin HbA1c [37]. In the kidneys, polyphenols reduce glomerular mesangial matrix dilation and fibrosis [38], inflammatory factors in diabetic and obese nephropathy [39], urea, creatinine, and plasma neutrophil gelatinase-associated lipocalin (NGAL) levels in rats with CKD [40]. They reduce the excretion of albumin in urine and increase creatinine clearance in rats with MetS [41]. The natural bioactive compounds can also prevent the development of AKI [42]. Oxidative stress also regulates the expression of matrix metalloproteinases (MMPs). Many studies have shown a significant relationship between polyphenols and the inhibition of MMPs [43–45]. MMPs are involved in the remodeling of the extracellular matrix and play an important role in renal diseases, including CKD and AKI. Taking into account the pathophysiological mechanisms of MetS and MetS-associated renal diseases, polyphenols are considered to be a very interesting potential protective and/or therapeutic candidate. Hence, the purpose of this research is the determination of the effect of polyphenolic extract from pomegranate peel on kidney damage in MetS.

## 2. Materials and Methods

### 2.1. Experimental Animals

**Figure 1:**
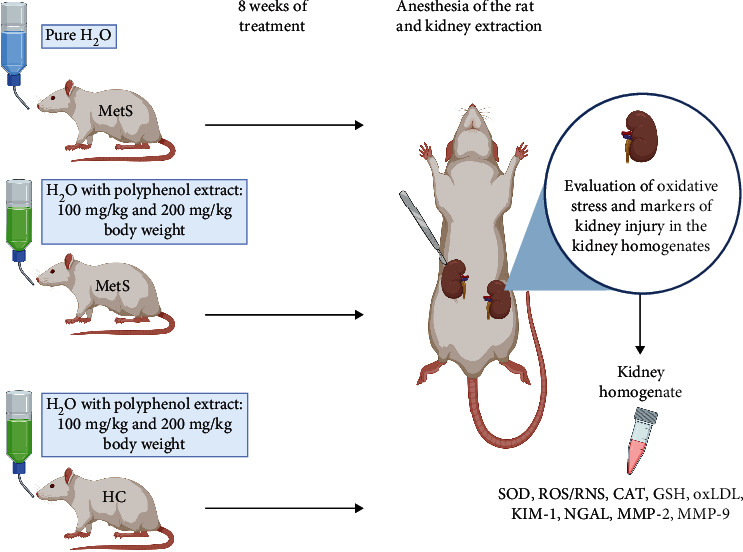
Scheme of the experiment, created with http://BioRender.com/.

Adult male Zucker Diabetic Fatty (ZDF-Leprfa/Crl) rats were obtained from Sulzfeld (Charles River Laboratories, Research Models and Services, Germany GmbH). The animals were divided into five groups of six rats each (Figure 1). All animals were fed the same diet (Purina LabDiet 5008, Charles River Laboratories, USA). The control group (MetS-Control) was rats with a leptin receptor missense mutation (ZDF fa/fa). The study group consisted of individuals with a mutation in the leptin receptor gene (ZDF fa/fa) and received polyphenol extract from pomegranate fruit peels mixed with water at a dose of 100 or 200 mg/kg body weight (MetS+100 or MetS+200). Two groups of healthy rats without MetS (ZDF fa/+) received the extract in the same doses (HC+100 and HC+200). The water with the extract was administered through a gastric tube for 8 weeks. The study was approved by the Ethics Committee for Experiments on Animals at the Ludwik Hirszfeld Institute of Immunology and Experimental Therapy, Polish Academy of Sciences, Wroclaw, Poland (Resolution 53/2017).

### 2.2. Kidney Isolation

Rats were sedated using a mixture of intramuscular anesthetics as follows: ketamine at a dose of 60 mg/kg and medetomidine at a dose of 0.3 mg/kg at week 8 of the experiment. The rats were euthanized via intraperitoneal injection of pentobarbital. The kidneys were rapidly excised from the animals. Organs were weighed and frozen in liquid nitrogen before preparing the homogenates.

### 2.3. Preparation of Kidney Homogenates

Frozen rat kidneys were crushed using a mortar and pestle in liquid nitrogen. Then, the kidneys underwent three cycles of freezing (in liquid nitrogen) and thawing (at 37°C) in a homogenization buffer (50 mmol/L Tris-HCl (pH 7.4), 3.1 mmol/L sucrose, 1 mmol/L dithiothreitol, 10 mg/mL leupeptin, 10 mg/mL soybean trypsin inhibitor, 2 mg/mL aprotinin, and 0.1% Triton X-100). After that, the kidney tissue was homogenized mechanically in an ice-cold homogenization buffer. The homogenate was centrifuged (10000 × *g* at 4°C for 15 minutes). Supernates were collected and stored at -80°C for further biochemical experiments.

### 2.4. Plant Material and Extract Preparation

Pomegranate (*Punica granatum L.*) is a rich source of polyphenols (mainly punicalagin) and has been widely used in traditional medicine. The polyphenolic extract was prepared from *Punica granatum L.* peels (cultivar Mollar de Eche) delivered from Spain. Dried peels of pomegranate fruit were extracted and reextracted twice with 50% ethanol. The obtained material was concentrated by a rotary evaporator (Rotavapor, Buchi Labortechnik AG, Switzerland) in a water bath at 40°C. Subsequently, the extract was adsorbed using Amberlite XAD-16 (Brenntag, Essen, Germany) resin, and after ethanol evaporation, the collected fraction was dried in an SPT-200 (Zeamil, Krakow, Poland) vacuum oven.

### 2.5. Identification and Quantification of Phenolic Compounds Using the LC-PDA–MS QTOF and UPLC-PDA Methods

Previously obtained polyphenol extracts from pomegranate peel were dissolved in MeOH/H_2_O/ascorbic acid (30 : 68 : 1 v/v/m) with 1% of a 37% hydrochloric acid mixture and performed on the ACQUITY Ultra Performance Liquid Chromatography (UPLC) system equipped with a photodiode array detector (PDA; Waters Corporation, Milford, MA) coupled to a quadrupole time-of-flight-mass spectrometry (QTOF-MS, Waters, Manchester, UK) with electrospray ionization (ESI) as a source operating in negative and positive ion modes with spectra acquired over a mass range from m/z 100 to 1800. Chromatographic separation was performed using UPLC BEH C18 columns (1.7 *μ*m, 2.1 mm × 100 mm; Waters Corporation, Milford, USA). A mobile phase flow rate of 0.42 mL/min throughout the gradient was used for elution. Water (acidified with 0.1% formic acid, v/v) and acetonitrile with 0.1% formic acid were used as mobile phases (A and B, respectively). The gradient was started with 99% of A in isocratic conditions for 1 minute, then an 11-minute linear gradient of 1% to 40% of B was applied. The B was increased to 100% at 12 to 14 minutes, and finally, returned to the initial conditions (99% of A) for 2 min as the reequilibration step. The optimal injected volume was 10 *μ*L. QTOF-MS experiments were performed in negative mode before and after fragmentation. Individual polyphenols were characterized by retention times and molecular masses. Collision-induced fragmentation experiments were performed using argon as the collision gas. The optimum values of the UPLC and QTOF-MS parameters were a capillary and cone voltage of 2500 V and 30 V, respectively. The capillary temperature was set to 300°C and the source heater temperature at 100°C with a drying gas (nitrogen) flow rate of 300 L/h. The results are presented in Table 1.

### 2.6. Measurement of Total Protein Concentration

The concentration of total protein in the kidney homogenates was measured by the Bradford method using Bio-Rad Protein Assay Dye Reagent (Bio-Rad) and a Spark Multimode Microplate Reader (Tecan Trading AG, Switzerland). Bovine serum albumin (BSA, heat shock fraction, ≥98%, Sigma-Aldrich) served as the protein standard.

### 2.7. Assessment of Superoxide Dismutase Activity

Total copper-zinc superoxide dismutase activity (SOD1) was measured in tissue homogenates with the help of the Superoxide Dismutase Assay Kit (Item no. 706002, Cayman Chemicals, Ann Arbor, MI, USA), which uses tetrazolium salt for detection of superoxide radicals. It is produced using xanthine oxidase and hypoxanthine. Collected homogenates were diluted 100-fold and assayed following the assay protocol. Absorbance was measured at 450 nm (Spark Multimode Microplate Reader, Tecan Trading AG, Switzerland). All measurements were performed in duplicate. Units of SOD1 activity were calculated from a standard curve using purified bovine erythrocyte SOD1 enzyme. The activity of SOD1 was expressed in U/mL. One unit of SOD1 activity is defined as the amount of enzyme needed to dismutase 50% of available superoxide radicals. The activity of SOD1 in kidney homogenate was converted to milligrams of total protein and expressed as U/mg protein.

### 2.8. Determination of Catalase Activity

Catalase (CAT) activity in kidney homogenates was determined by the ultraviolet (UV) spectrophotometric method of Aebi. Each sample of tissue lysate was diluted 501-fold (10 *μ*L sample was mixed with 5 mL of phosphate buffer and kept on ice). A sodium, potassium phosphate buffer (50 mM, pH 7.4), was prepared by dissolving 6.81 g of KH_2_PO_4_ (Cat. no. 117420202, Chempur, Poland) and 8.90 g of Na_2_HPO_4_ (Cat. no. 117992801, Chempur, Poland) in 1000 mL of distilled water. The solutions were mixed using the proportion of 1 : 1.5 (v/v). In a quartz cuvette containing 1000 *μ*L of diluted kidney homogenate, 500 *μ*L of 20 mM H_2_O_2_ in phosphate buffer was added, and the decrease in absorbance was read at 240 nm for 30 seconds in a spectrophotometer (UV-Vis Double Beam HALO DB-20 Spectrofotometer, Dynamica GmbH, Switzerland). The 20 mM H_2_O_2_ solution was freshly prepared from 3% H_2_O_2_ (Galfarm, Poland) and standardized using a molar extinction coefficient (*ε* = 43.6 M^–1^ cm^–1^ at 240 nm). This solution was used to determine CAT activity. All samples were assessed in triplicate. One unit of CAT will decompose 1.0 micromole of hydrogen peroxide to oxygen and water per minute at a pH of 7.0 and temperature of 25°C with a hydrogen peroxide substrate concentration of 20 mM.

### 2.9. Determination of Glutathione Concentration

A Glutathione (GSH) Assay Kit (Item no. 703002, Cayman Chemical Company, Ann Arbor, MI, USA) was used to measure GSH levels in kidney homogenate. The reaction between sulfhydryl groups of GSH and Ellman's reagent (DTNB, 5,5′-dithio-bis-2-nitrobenzoic acid) results in the yellow-colored product 5-thio-2-nitrobenzoic acid (TNB). Samples were diluted 100-fold. The absorbance of TNB was measured at 410 nm using a microplate reader (Spark Multimode Microplate Reader, Tecan Trading AG, Switzerland). All measurements were performed in duplicate. The GSH concentration was expressed in *μ*M/mg protein.

### 2.10. Reactive Oxygen and Nitrogen Species in Kidney Homogenates

The OxiSelect™ In Vitro ROS/RNS Assay Kit (Cell Biolabs, San Diego, USA) was used to determine the oxidative stress in kidney homogenates. The assay measures total ROS and RNS, including hydrogen peroxide (H_2_O_2_), nitric oxide (NO), peroxyl radical (ROO^•^), and peroxynitrite anion (ONOO^−^), using a fluorogenic probe, dichlorodihydrofluorescin DiOxyQ (DCFH-DiOxyQ). The probe was primed with a quench removal reagent for the highly reactive DCFH form. In this reactive state and in the presence of ROS and RNS, the DCFH is rapidly oxidized to the highly fluorescent 2′,7′-dichlorodihydrofluorescein (DCF). Fluorescence intensity is proportional to total ROS/RNS levels within the sample. The total ROS/RNS level was normalized to the total protein concentration.

### 2.11. Oxidized LDL (OxLDL) Concentration

OxLDL concentrations in tissue homogenates were determined using the ELISA method (Item no. E0527r, Human OxLDL ELISA Kit, EIAab Science Inc., Wuhan, China). Analysis was conducted in duplicate. The microplate was precoated with an anti-oxLDL antibody. Samples were added to the wells with a biotin-conjugated antibody preparation specific for the antigen, and then, avidin conjugated to horseradish peroxidase was added. Then, a 3,3′,5,5′-tetramethylbenzidine (TMB) was added to each well. The enzyme-substrate reaction was terminated by the addition of a sulphuric acid solution. The color change was measured spectrophotometrically at a wavelength of 450 nm (Spark Multimode Microplate Reader, Tecan Trading AG, Switzerland). The oxLDL concentration was expressed in ng/mg protein.

### 2.12. MMP-2 and MMP-9 Concentrations in Kidney Homogenates

MMP-2 and MMP-9 concentrations in kidney homogenates were measured using quantitative Quantikine ELISA Assay for Total MMP-2 and Rat Total MMP-9 (R&D Systems, Minneapolis, MN, USA) according to the manufacturer's instructions. MMP-2 and MMP-9 were immobilized with monoclonal antibodies specific to the protein and were detected with the use of an anti-Total-MMP-2 and anti-Total-MMP-9 polyclonal antibody conjugated to streptavidin-horseradish (HRP) peroxidase. Next, TMB substrate solution was added to the reaction. As a stop solution, sulphuric acid was used. The minimum detectable dose of the test was 0.033 ng/mL for MMP-2 and 0.013 ng/mL for MMP-9.

### 2.13. NGAL in Kidney Homogenates

NGAL concentrations in kidney homogenates were measured using the quantitative ELISA Assay for NGAL (EIAab Science Inc., Wuhan, China) according to the manufacturer's instructions. NGAL immobilized with a monoclonal antibody specific to this protein was detected using a biotin-conjugated anti-NGAL polyclonal antibody. Then, avidin conjugated to horseradish peroxidase was added. Next, TMB substrate solution was added to develop the reaction. As a stop solution, sulphuric acid was used. The minimum detectable dose of the test was 0.460 ng/mL.

### 2.14. Kidney Injury Molecule-1 Concentration

Kidney injury molecule-1 (KIM-1) concentrations in kidney homogenates were measured using the quantitative ELISA Assay for KIM-1 (EIAab Science Inc., Wuhan, China) according to the manufacturer's instructions. Similar to the NGAL assay, KIM-1 was immobilized with a monoclonal antibody specific to this protein and was detected with the use of a biotin-conjugated anti-NGAL polyclonal antibody. Then, avidin conjugated to horseradish peroxidase was added. Next, TMB substrate solution was added to develop the reaction. As a stop solution, sulphuric acid was used. The minimum detectable dose of the test was 0.089 ng/mL.

### 2.15. Statistical Analysis

The experimental data were analyzed using GraphPad Prism 8.0.1 for Windows (GraphPad Software, San Diego, California USA). The Shapiro-Wilk normality test was used to assess the normality of variance changes. The Student's *t*-test or Mann–Whitney *U* test was used for comparisons between two groups of measurement data. Results were expressed as mean ± SEM, and a *p* value of < 0.05 was regarded as statistically significant.

## 3. Results

### 3.1. The Influence of Polyphenolic Extract on Oxidative Status in Kidney Tissue

**Figure 2:**
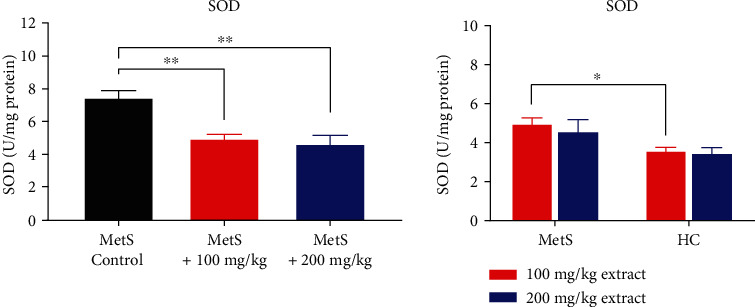
SOD activity in kidney tissue (^∗^*p* < 0.05 and ^∗∗^*p* < 0.01).

**Figure 3:**
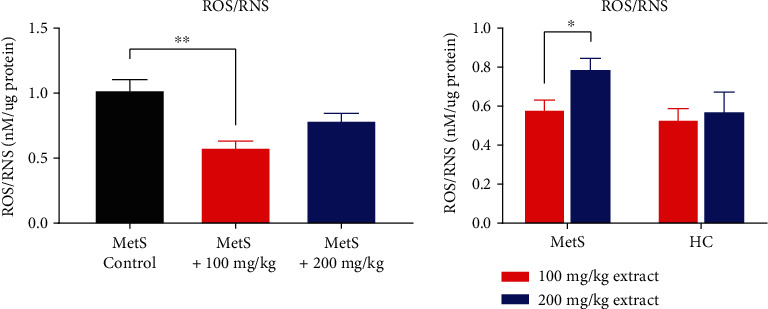
ROS/RNS concentration in kidney tissue (^∗^*p* < 0.05 and ^∗∗^*p* < 0.01).

Pomegranate peel polyphenols have a pronounced effect on the activity of SOD (Figure 2) and the concentration of ROS/RNS in kidney tissue (Figure 3). SOD activity in the kidneys of rats receiving 100 mg/kg and 200 mg/kg of polyphenolic extract was significantly lower than in subjects with MetS and receiving a standard diet (4.89 ± 0.80 U/mg, *p* = 0.0087 and 4.53 ± 1.41, *p* = 0.0043 vs. 7.38 ± 1.21 U/mg, respectively). There were no differences between the doses. Healthy animals receiving the extract in identical doses had lower SOD activities than animals with a mutation in the leptin gene treated with polyphenols; however, the difference was statistically significant only in the group receiving 100 mg/kg of extract (3.51 ± 0.51 U/mg vs. 4.89 ± 0.80 U/mg, *p* = 0.015 and 3.44 ± 0.64 U/mg vs. 4.53 ± 1.41, *p* = 0.420 for 200 mg/kg of polyphenols, respectively). This indicates that the polyphenolic extract (200 mg/kg and 100 mg/kg) significantly reduces SOD activity.

Lower extract doses led to a significant reduction in ROS/RNS concentration (Figure 3) (0.58 ± 0.11 nM/*μ*g vs. 1.03 ± 0.18 nM/*μ*g, *p* = 0.008). The dose of 200 mg/kg reduced the ROS/RNS concentration, but the reduction was greater at the lower dose (0.79 ± 0.12 nM/*μ*g vs. 1.03 ± 0.18 nM/*μ*g, *p* = 0.055). No differences in ROS/RNS concentrations were found between healthy and MetS animals after the use of polyphenols (0.53 ± 0.12 nM/*μ*g vs. 0.58 ± 0.11 nM/*μ*g, *p* = 0.421 for HC+100 vs. MetS+100 and 0.57 ± 0.22 nM/*μ*g vs. 0.79 ± 0.12 nM/*μ*g, *p* = 0.222 for HC+200 vs. MetS+200). MetS rats had significantly higher kidney ROS/RNS concentrations (0.79 ± 0.12 nM/*μ*g vs. 0.58 ± 0.11 nM/*μ*g, *p* = 0.039) after using the higher dose of the extract. This shows that a low dose of the extract has an antioxidant effect, and a high dose may have prooxidative properties.

**Figure 4:**
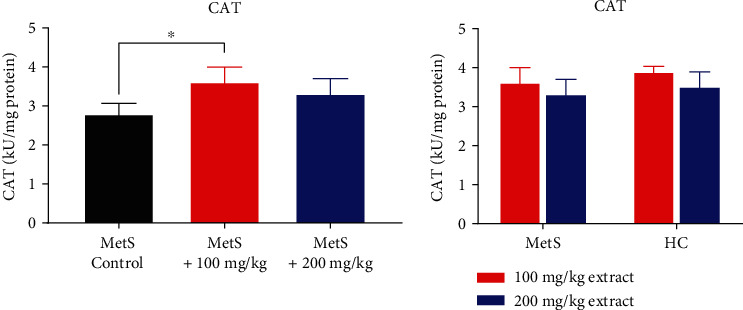
CAT activity in kidney tissue (^∗^*p* < 0.05).

CAT activity was higher in rats with MetS receiving polyphenolic extract than in animals only with MetS, but only subjects in the MetS+100 group had a borderline value of statistical significance (3.62 ± 0.93 kU/mg vs. 2.79 ± 0.61 kU/mg, *p* = 0.051 for MetS+100 group and 3.31 ± 0.86 kU/mg vs. 2.79 ± 0.61 kU/mg, *p* = 0.421 for MetS+200 group, Figure 4). There was no significant difference in CAT activity at the polyphenol doses between MetS and healthy animals (3.62 ± 0.94 kU/mg and 3.90 ± 0.29 kU/mg for MetS+100 vs. HC+100, *p* = 0.609 and 3.31 ± 0.86 kU/mg and 3.51 ± 0.85 kU/mg for MetS+200 vs. HC+200, *p* = 0.547). However, there is a tendency for decreased CAT activity after using a higher dose of the extract.

**Figure 5:**
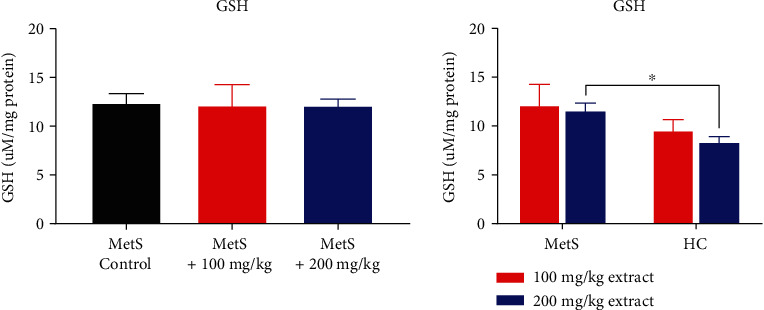
GSH concentration in kidney tissue (^∗^*p* < 0.05).

No differences in GSH concentrations were found after polyphenol administration in MetS animals (12.17 ± 4.69 *μ*M/mg vs. 12.42 ± 2.09 *μ*M/mg, *p* = 0.548 and 12.15 ± 1.41 *μ*M/mg vs. 12.42 ± 2.09 *μ*M/mg, *p* = 0.841 for 100 mg/kg and 200 mg/kg of polyphenols, respectively, Figure 5). In the group of animals treated with polyphenols, a lower concentration of GSH was observed in healthy rats compared to animals with MetS after the use of the extract at a dose of 100 mg/kg (8.82 ± 2.10 *μ*M/mg vs. 12.17 ± 4.69 *μ*M/mg, *p* = 0.151) and a statistically significant lower concentration of GSH at the dose of 200 mg/kg (8.36 ± 1.25 *μ*M/mg vs. 12.15 ± 1.41 *μ*M/mg, *p* = 0.016).

### 3.2. The Influence of Polyphenolic Extract on Lipid Peroxidation

**Figure 6:**
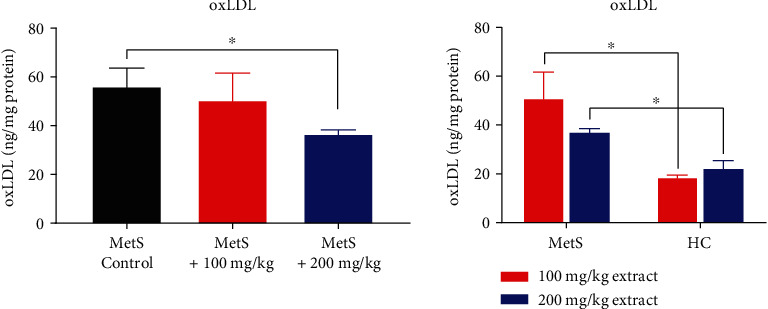
OxLDL concentration in kidney tissue (^∗^*p* < 0.05).

Polyphenolic extract at a dose of 200 mg/kg significantly decreased the concentration of oxLDL in animals with MetS (36.77 ± 3.62 ng/mg vs. 58.49 ± 20.62 ng/mg, *p* = 0.016, Figure 6). However, this effect was not observed at the dose of 100 mg/kg (50.63 ± 26.98 ng/mg vs. 58.49 ± 20.62 ng/mg, *p* = 0.662). Significant differences were also observed between healthy animals receiving the extract and those with MetS. Healthy rats had significantly lower oxLDL concentrations (18.04 ± 2.29 ng/mg vs. 50.63 ± 26.98 ng/mg, *p* = 0.009 for HC+100 vs. MetS+100 and 22.06 ± 7.24 ng/mg vs. 36.77 ± 3.62 ng/mg, *p* = 0.016 for HC+200 vs. MetS+200).

### 3.3. The Influence of Polyphenolic Extract on KIM-1 and NGAL

**Figure 7:**
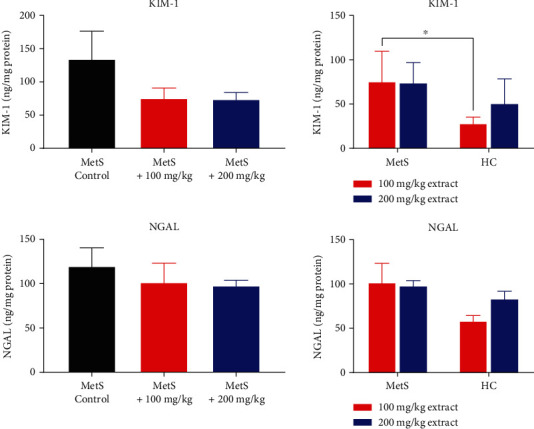
Concentrations of KIM-1 and NGAL in kidney tissue (^∗^*p* < 0.05).

Polyphenol extract did not lower the concentration of kidney damage markers (KIM-1 and NGAL) in MetS kidneys. However, the level of KIM-1 was higher in the MetS group. There were no statistically significant changes in the concentration of KIM-1 in rats treated with the polyphenol extract compared to the untreated group (Figure 7). However, there was a trend in the reduction of KIM-1 concentration for both doses of the extract (75.54 ± 33.98 ng/mg vs. 134.54 ± 93.23 ng/mL, *p* = 0.310 for MetS+100 vs. MetS-Control and 73.92 ± 22.85 ng/mL vs. 134.54 ± 93.23 ng/mL, *p* = 0.421 for MetS+200 vs. MetS-Control). Healthy animals from the HC+100 group had a significantly lower concentration of KIM-1 than those in the MetS+100 group (28.26 ± 7.04 ng/mg vs. 73.92 ± 22.85 ng/mL, *p* = 0.032).

Pomegranate peel extract did not significantly change the concentration of NGAL, regardless of the dose (101.67 ± 48.40 ng/mg vs. 119.88 ± 46.09 ng/mg, *p* = 0.691 MetS+100 vs. MetS-Control and 97.77 ± 13.62 ng/mg vs. 119.88 ± 46.09 ng/mg, *p* = 0.841 for MetS+200 vs. MetS-Control, Figure 7). Healthy animals have a slightly lower concentration of NGAL, especially in the HC+100 group.

### 3.4. The Influence of Polyphenolic Extract on MMPs

**Figure 8:**
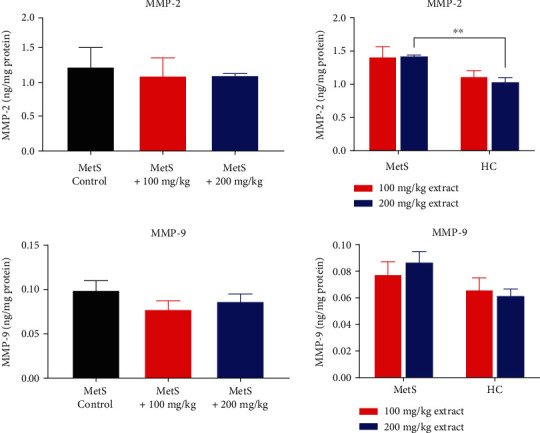
Concentration of MMP-2 and MMP-9 in kidney tissue (^∗∗^*p* < 0.01).

The administration of polyphenol extract to MetS animals did not affect the concentrations of MMP-2 and MMP-9 in kidney tissue (Figure 8). Only healthy rats had a lower concentration of MMP-2 compared to MetS animals receiving an extract dose of 200 mg/kg (1.04 ± 0.14 ng/mg vs. 1.43 ± 0.03 ng/mg, *p* = 0.008).

## 4. Discussion

**Figure 9:**
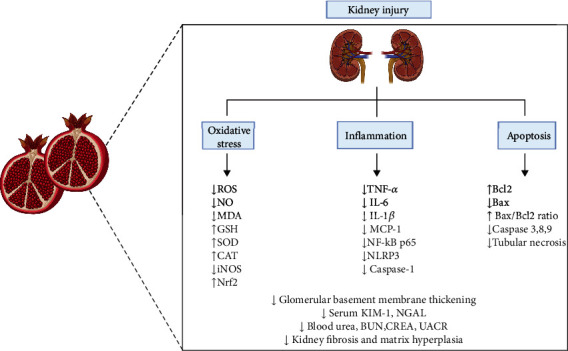
The positive effect of punicalagin on kidney injury [46–49, 50, 51] created with http://BioRender.com/.

The use of *Punica granatum L.* extract has gained interest thanks to its antioxidative and anti-inflammatory properties (Figure 9) [46–51]. Almost half of a pomegranate fruit's weight is from the peel. The peel is rich in bioactive substances such as vitamins, flavonoids, phenolics, ellagitannins, and minerals [52]. Different products derived from pomegranates such as extracts, oils, and juices have been confirmed to positively affect MetS, diabetes, and comorbid diseases [53]. Pomegranate has the ability to reduce insulin resistance via the peroxisome proliferator-activated receptor gamma (PPAR*γ*) and therefore regulates abnormal oxidative stress in macrophages and inhibits pathological cardiac changes. In addition, pomegranate and punicalagin improve lipid metabolism [53, 54]. Pomegranate extract administration can reduce total cholesterol and triglyceride levels [53]. Treatment with the extract has a positive effect on the pathologic accumulation of atherosclerotic lesions by reducing macrophage oxLDL uptake [54]. Punicalagin has been proven to activate the forkhead box O1 (FoXO1) pathway, which is thought to prevent mitochondrial loss and vascular disturbance. Moreover, punicalagin downregulates the expression of IL-6 and MMP-1 which contributes to inflammation [55]. Punicalagin is proved to regulate cell death in particular apoptosis and pyroptosis. The treatment with punicalagin decreases Bax (proapoptotic factor) and upregulates Bcl-2 (antiapoptotic factor) which led to downregulation of caspases involved in apoptotic cell death—caspase 3,8 and 9 [50]. Besides, punicalagin mitigate pyroptosis via HMGB-1/TLR4/NF-*κ*B signaling pathway by downregulation of the expression of involved proteins [46]. In this study, we evaluated the positive effects of *Punica granatum L.* polyphenol-rich extract on oxidative stress and kidney injury in MetS. We showed that MetS contributes to kidney injury, and antioxidants can reduce this damage. Moreover, we confirmed that the administration of *Punica granatum L.* extract has positive effects on the reduction of oxidative stress and changes in the activity of antioxidative enzymes. Previously, it has been reported that MetS correlates with increased oxidative stress and lower antioxidative defense capacity due to the reduction of antioxidant enzyme levels [37]. Obesity and diabetes influence kidney functioning and may induce AKI [56, 57]. So far, pathophysiological mechanisms are not well defined; however, multiple factors have been proposed as predictors of the development of AKI and its progression into CKD. Among these, glomerulopathy, inflammation, dysfunction of the endothelium, activation of the sympathetic nervous system, and renin-angiotensin-aldosterone system (RAAS) are mentioned [58]. Hemodynamic changes such as an increased GFR and albumin excretion accompanied by glomerulopathy led to podocyte cell damage and mesangial enlargement [58]. Obese patients and patients suffering from diabetes often demonstrate hypertension that disturbs the normal blood flow through the kidneys [57]. Besides, oxidative stress is proposed as an important risk factor for AKI development and its progression [58]. Downregulation of the endogenous antioxidant system and increased production of ROS correlate with the levels of kidney injury markers (KIM-1, NGAL) and renal tubular cell injury [50]. For this reason, naturally existing polyphenols have been investigated as a potential protector from AKI and CKD, due to their ability to reduce inflammation and oxidative stress which are the main triggers of kidney dysfunction [50, 59].

SOD is an enzyme that catalyzes the conversion of superoxide into molecular oxygen and hydrogen peroxide. We observed that higher SOD activity was found in the kidneys of MetS animals, and treatment with *Punica granatum L.* extract reduced the activity. Punicalagin reduces ROS generation and exhibits strong superoxide radical scavenging activity [60]. We have found that a low concentration of pomegranate extract can reduce ROS/RNS levels in kidney tissue. Reducing oxidative stress by the direct scavenging of ROS/RNS in the kidneys can lead to a decrease in SOD activity. A number of MetS risk factors, such as fasting glucose levels, waist circumference, triglycerides, high-density lipoprotein cholesterol (HDL-C) serum levels, high blood pressure, and previous treatment for hypertension might affect the antioxidant enzyme systems. The LIPGENE study showed that patients with 3, 4, or 5 MetS factors had higher SOD levels than those with only two [61]. Therefore, SOD can be upregulated by high concentrations of ROS and increased oxidative stress. This could explain our findings, that SOD activity was the highest in the MetS groups which correlate with higher ROS/RNS concentrations. Surprisingly, many of the ellagitannins exhibited a relatively high prooxidant activity. Punicalagin shows antioxidative activity in the prooxidant activity test [62]. In our study, the higher concentration of the extract corresponded with higher ROS/RNS levels compared to the lower concentration in the MetS rates. This indicates the prooxidative effects of the extract when used at higher doses. In another study, a polyphenol-rich diet has been confirmed to positively affect SOD activity and the expression of NF-E2-related factor 2 (Nrf2) which is a transcription factor that regulates antioxidant enzyme activities. Besides, the kidneys of animals with MetS on a blueberry-enriched diet demonstrated fewer pathological changes and organ fibrosis [63]. Interestingly, the diet significantly increased both SOD and CAT activity, indicating a beneficial antioxidant effect in MetS animals [63]. In our study, the use of polyphenols was associated with a decrease in SOD activity and ROS/RNS concentrations.

CAT is the second most important enzyme in the antioxidative system. CAT plays a crucial role in the reduction of hydrogen peroxide. In our study, CAT activity in the kidney tissue homogenates was higher in both groups treated with polyphenolic extract, but a significant increase was only found after using the concentration of 100 mg/kg. There is a tendency for pomegranate extract to upregulate CAT activity, but the higher dose was less effective. This showed an increase in antioxidant activity to values close to those occurring in healthy rats receiving the extract.

The dysregulation of ROS is well-known in diabetic kidney disease (DKD). DKD impairs normal mitochondrial function, including mitochondrial biogenesis, fission, and fusion [64]. But also metabolic changes and the above-mentioned Nrf2 play a major role in the etiology and pathogenesis of DKD. Renal exposure to high blood glucose levels is associated with an overproduction of ROS and a reduction of antioxidants. Hyperglycemia-induced ROS production leads to the increased formation of advanced glycation end products (AGEs) and activation of nuclear factor *κ*B (NF*κ*B) pathways which lead to abnormal endothelial and vascular cell activity [65]. Oxidative stress induces cellular apoptosis, glomerular distortion, and the regression of foot processes in podocytes, with consequent loss of integrity of the glomerular barrier. These abnormalities overlap other vascular alterations associated with persistently high levels of ROS [66].

We did not detect any significant changes in GSH concentrations in the kidneys of rats with MetS, not treated and treated with polyphenol-rich extract, indicating that the extract did not affect GHS concentrations in MetS animals. However, we observed a higher concentration of GSH in kidneys treated with extract in MetS in comparison to HC. An adequate supply of GSH in the kidneys is very important to maintain normal functioning and protection against oxidative stress. It is associated with an increased aerobic metabolism and kidney exposure to high concentrations of various oxidants [67]. Renal GSH metabolism is one of the most important aspects in the study of kidney function in MetS. Downregulation of GSH biosynthesis contributes to oxidative stress and kidney injury. Intracellular thiol concentrations, which control key molecular mechanisms of cells, are especially important in patients suffering from CKD [68]. One study indicated that GSH significantly suppressed the diabetes-induced increase in urinary 8-hydroxy-2′-deoxyguanosine, albumin, and creatinine levels. The authors concluded that GSH treatment can beneficially affect diabetic rats and suggests the usefulness of dietary GSH treatment to reduce diabetic complications [69].

A high concentration of ROS/RNS leads to the oxidation of proteins and lipids. OxLDL is an indicator of abnormal lipid modification and oxidative stress. The high concentrations of LDL and oxLDL, found in MetS, may induce pathological changes in the glomerulus [70]. The accumulation of circulating LDL in the glomeruli provokes mesangial cell proliferation and renal impairment followed by glomerulosclerosis [70]. OxLDL activates apoptosis and the progression of renal damage [71]. Also, it stimulates the oxLDL receptor, CD36 expression, and activation of Nrf2, which initiates the prooxidative pathway and contributes to kidney fibrosis [70, 72]. Our findings confirmed that oxLDL concentrations were higher in the groups with MetS compared to the corresponding healthy groups. Furthermore, treatment with high-concentration pomegranate polyphenolic extract significantly reduced the tissue levels of oxLDL. Pomegranate juice has previously been shown to reduce the uptake of oxLDL by macrophages and the biosynthesis of cholesterol [63, 73]. Reduced oxidative stress and its consequences regulate the formation of foam cells and atherosclerosis [63, 73]. High levels of LDL and oxLDL may induce pathological changes in the glomerulus, the most vulnerable part of the nephron [70].

KIM-1 is a biomarker of kidney injury since its expression is very low in healthy kidneys and is expressed by cells of the proximal tubule. It is a transmembrane glycoprotein that is rapidly upregulated and secreted into the urine after the damaging acts [74]. However, the progression of diabetes-related kidney disease and kidney fibrosis results in a reduction in KIM-1 expression and urinary levels [10]. NGAL is a small protein that belongs to the lipocalin family. It is physiologically produced at a low level by various organs, such as the kidneys, lungs, trachea, stomach, and colon [61]. Harmful conditions such as ischemic and toxic AKI as well as diabetic nephropathy increase both the expression of KIM-1 and NGAL [22, 61, 75]. KIM-1 and NGAL expression and serum levels are also elevated in diabetic nephropathy [50]. We found a tendency for animals given the polyphenolic extract in both doses had a lower concentration of KIM-1. In addition, significantly lower concentrations of KIM-1 were found in the kidney tissue homogenates of healthy rats treated with 100 mg/kg of extract compared to the corresponding group of rats with MetS (MetS+100 mg/kg). We noticed no significant difference in NGAL concentrations between the groups. In other studies, treatment with pomegranate extract in AKI reduces the expression and serum concentration of KIM-1 and NGAL. Pomegranate due to its antioxidative effects can reduce serum levels of injury markers during gentamicin-associated nephrotoxicity, which corresponded to the histological changes in the kidney structure [75]. Curcumin, a natural strong antioxidant has been proven to positively affect kidney functioning by reducing cell death [59]. However, curcumin reduces the expression of kidney injury markers and their levels in the urine of diabetic rats [59]. It has been demonstrated that high punicalagin concentrations cause liver injury and fibrosis [51], as well as damage to the kidney structure in cattle following *Terminalia oblongata* combustion [76]. This could explain our findings that high concentrations of pomegranate extract were associated with higher levels of kidney injury markers in the healthy group treated with 200 mg/kg of extract.

MMPs are a class of proteins that degrade extracellular matrix components and contribute to the physiological and pathophysiological remodeling of tissues including the kidney [76]. MMPs are thought to play an important role in the progression of kidney disease leading to kidney fibrosis and inflammation. Gelatinases (MMP-2 and MMP-9) are members of the MMP family that degrades collagen and other structural proteins frequently found in kidney cells. The activity is controlled at several stages including protein expression, posttranslational modifications, and through specific inhibitors [77]. High expression and activation of the gelatinases MMP-2 and MMP-9 lead to the epithelial-mesenchymal transition of tubular cells and changes in the basement membrane. As a result, mesenchymal cells migrate and form an abnormal fibrotic core [78]. MMP-2 and MMP-9 activity can also be activated by increased oxidative stress which plays an important role in the pathogenesis of CKD [79]. Inhibition of MMP-2 has been demonstrated to have positive effect on heart ischemia-reperfusion injury [80]. Tang et al. demonstrated that punicalagin can reduce the activity of MMP-2 and MMP-9 in HeLa cells while increasing the expression of tissue inhibitors of metalloproteinases 1 (TIMP-1) and TIMP-3 [81]. In our study, we noted that MMP-2 concentrations in the kidney were significantly higher in the MetS group treated with high concentrations of polyphenol extract (200 mg/kg) compared to the corresponding healthy group. This might be a warning sign for the progression of abnormal changes in the kidneys of rats with MetS.

It has been reported that patients with MetS have a higher blood viscosity [82]. In MetS, oxidative stress and chronic systemic inflammation increase blood viscosity by decreasing erythrocyte deformability and the rheological properties of blood [83]. Obese patients with metabolic disorders are at great risk of developing hyperviscosity syndrome (HVS) which alters tissue perfusion and can lead to complications such as nephropathy, AKI, and glucose intolerance [82]. Impaired kidney function and AKI are generally an effect of tubular obstruction and renal ischemia. The use of natural antioxidants, e.g., punicalagin or berberine, may be of benefit in the treatment of AKI [22, 84].

## 5. Limitations

Our study has a few limitations. First, there is no control group of healthy rats, which should be watered only with pure water without polyphenolic extract. Second, the sample size was limited since there are only 6 animals in each group. Moreover, we suggest that further study should be performed to provide information about kidney morphological changes and various parameters in the urine, and blood should be evaluated.

## 6. Conclusions

This study provides evidence about the increased oxidative stress in MetS kidneys. We detected that *Punica granatum L.* extract may have two opposite effects: antioxidative and prooxidative, and the effect is related on the extract concentration. Moreover, we showed that *Punica granatum L.* extract has a positive effect on the kidneys by regulating ROS/RNS production and modifying the endogenous antioxidative system. This experiment allowed us to investigate the effect of different bioactive substances of pomegranate peel and not just one particular polyphenol to kidney damage associated with the metabolic syndrome. Treatment with polyphenolic extract may be beneficial for MetS kidneys prone to AKI and structural damage.

## Figures and Tables

**Table 1:** Mass spectrometry (MS) characteristics and the content of phenolic compounds in pomegranate peel extract. Ranked by retention time (Rt).

Rt	MS [M-H]^−^ (m/z)	MS/MS [M-H]^−^ (m/z)	Name of compound	Polyphenol content
1.67	331	271/169	Galloyl-glucose	2.00 ± 0.03
1.73	781	721/601	Punicalin *α*/A	3.11 ± 0.06
2.02	1083	611/331/146	HHDP-gallagyl-hexoside (punicalagin)	4.20 ± 0.09
2.12	1083	781/622/301	Punicalagin isomer	14.82 ± 1.04
2.33	933	631/450/301	Ellagitannin	4.71 ± 0.40
2.87	1083	781/301	HHDP-gallagyl-hexoside (punicalagin)	93.91 ± 2.05
3.12	1085	907/783/301	Ellagic acid derivative	2.49 ± 0.53
3.69	1083	781/301	HHDP-gallagyl-hexoside (punicalagin)	157.0 ± 2.65
3.89	799	301	Granatin A	4.74 ± 0.32
5.08	783	481/301	Ellagitannin	25.86 ± 1.53
6.20	1085	933/301	Digalloyl-gallagyl-hexoside	10.37 ± 0.65
6.25	783	481/301	Ellagitannin	13.51 ± 0.99
6.38	463	301	Ellagic acid-hexoside	33.63 ± 1.23
6.89	951	907/635/301	Galloyl-HHDP-DHHDP-hex (granatin B)	2.68 ± 0.11
			Total (mg/g dw)	373.05

## Data Availability

The data that support the findings of this study are openly available at the request of the reader.
